# Invasive *Streptococcus pneumoniae* infection among hospitalized patients in Jingzhou city, China, 2010-2012

**DOI:** 10.1371/journal.pone.0201312

**Published:** 2018-08-20

**Authors:** Hui Jiang, Yang Huai, Hui Chen, Timothy M. Uyeki, Maoyi Chen, Xuhua Guan, Shali Liu, Youxing Peng, Hui Yang, Jun Luo, Jiandong Zheng, Jigui Huang, Zhibin Peng, Nijuan Xiang, Yuzhi Zhang, John D. Klena, Dale J. Hu, Jeanette J. Rainey, Xixiang Huo, Lin Xiao, Xuesen Xing, Faxian Zhan, Hongjie Yu, Jay K. Varma

**Affiliations:** 1 Key Laboratory of Surveillance and Early-warning on Infectious Disease, Division of Infectious Disease, Chinese Center for Disease Control and Prevention, Beijing, China; 2 China-US Collaborative Program on Emerging and Re-Emerging Infection Disease, Center for Global Health, Centers for Disease Control and Prevention, Beijing, China; 3 Hubei Provincial Center for Disease Control and Prevention, Wuhan, China; 4 Influenza Division, National Center for Immunization and Respiratory Diseases, Atlanta, GA, United States of America; 5 Jingzhou Center for Disease Control and Prevention, Jingzhou, China; 6 Jingzhou Central Hospital, Jingzhou, China; 7 Jingzhou First People’s Hospital, Jingzhou, China; 8 Jingzhou Second People’s Hospital, Jingzhou, China; 9 Jingzhou Maternal and Children’s Hospital, Jingzhou, China; 10 Global Disease Detection Branch, Division of Global Health Protection, Center for Global Health, Centers for Disease Control and Prevention, Atlanta, GA, United States of America; Public Health England, UNITED KINGDOM

## Abstract

**Background:**

*Streptococcus pneumoniae (Sp)* is a leading cause of bacterial pneumonia, meningitis, and sepsis and a major source of morbidity and mortality worldwide. Invasive pneumococcal disease (IPD) is defined as isolation of *Sp* from a normally sterile site, including blood or cerebrospinal fluid. The aim of this study is to describe outcomes as well as clinical and epidemiological characteristics of hospitalized IPD case patients in central China.

**Methods:**

We conducted surveillance for IPD among children and adults from April 5, 2010 to September 30, 2012, in four major hospitals in Jingzhou City, Hubei Province. We collected demographic, clinical, and outcome data for all enrolled hospitalized patients with severe acute respiratory infection (SARI) or meningitis, and collected blood, urine, and cerebrospinal fluid (CSF) for laboratory testing for *Sp* infections. Collected data were entered into Epidata software and imported into SPSS for analysis.

**Results:**

We enrolled 22,375 patients, including 22,202 (99%) with SARI and 173 (1%) with meningitis. One hundred and eighteen (118, 3%) with either SARI or meningitis were *Sp* positive, 32 (0.8%) from blood/CSF culture, and 87 (5%) from urine antigen testing. Of those 118 patients, 57% were aged ≥65 years and nearly 100% received antibiotics during hospitalization. None were previously vaccinated with 7-valent pneumococcal conjugate vaccine (PCV 7), 23-valent pneumococcal polysaccharide vaccine, or seasonal influenza vaccine. The main serotypes identified were 14, 12, 3, 1, 19F, 4, 5, 9V, 15 and 18C, corresponding to serotype coverage rates of 42%, 63%, and 77% for PCV7, PCV10, and PCV13, respectively.

**Conclusions:**

Further work is needed to expand access to pneumococcal vaccination in China, both among children and potentially among the elderly, and inappropriate use of antibiotics is a widespread and serious problem in China.

## Introduction

*Streptococcus pneumoniae* (*Sp*) is a leading cause of bacterial pneumonia, meningitis, and sepsis and a major source of morbidity and mortality worldwide[1]. Although pneumococcal vaccines help prevent pneumococcal disease, the WHO estimates that approximately 1 million children die of pneumococcal disease every year, mostly in developing countries[2, 3]. Although global estimates are valuable, national public health officials need country and region-specific estimates of disease incidence and prevalence for policy decision making[4]. To date, China has lacked substantial, high quality surveillance systems to measure incidence of *Sp* infection associated with respiratory disease and meningitis.

Bacterial culture from blood or cerebrospinal fluid is the established method of diagnosing invasive *Sp* infection[5], but bacterial culture is infrequently used in China hospitals. Additionally, patients in China frequently take antibiotics by self-purchased before seeking medical care[6], potentially resulting in false negatives for *Sp* culture. The above factors make it challenging to estimate the burden of *Sp* infections in China.

In April 2010, the Chinese Center for Disease Control and Prevention (China CDC), in collaboration with the United States Centers for Disease Control and Prevention (US CDC), launched active surveillance for severe acute respiratory infection (SARI) and meningitis in Jingzhou city, Hubei province. The aim of this project was to characterize etiologies of SARI and meningitis in people admitted with these conditions to Jingzhou City hospitals. We analyzed data from this surveillance system to describe the epidemiology and clinical characteristics of SARI and meningitis patients with *Sp* infections.

## Methods

We conducted surveillance in three general hospitals and one pediatric hospital in Jingzhou City, Hubei Province located in central China as previously described[7]. We collected demographic, clinical, and outcome data for all enrolled hospitalized patients with severe acute respiratory infection (SARI) or meningitis. Additionally, we obtained blood, urine, and cerebrospinal fluid (CSF) samples to test for *Sp* infections between April 5, 2010 and September 30, 2012.

### Case definitions

Patients met the case definition for SARI if they were hospitalized and had: temperature ≥37.3°C and at least one of the following: cough, sore throat, tachypnea, difficulty breathing, abnormal breath sounds on auscultation, sputum production, hemoptysis, chest pain, or chest radiograph consistent with pneumonia. Patients met the case definition for meningitis if they were hospitalized and had: temperature ≥37.3°C and change in mental status, meningeal irritation signs (positive Kernig’s or Brudzinski’s signs), bulging fontanelle (if aged <12 months), toxic appearance, petechial rash, or purpural rash. Patients were excluded from the study if they were infants who were born in the hospital and became ill while hospitalized.

A case was defined as laboratory-confirmed for *Sp* if it had a blood, urine (≥18 years), or CSF specimen that was positive for *Sp* by bacterial culture or antigen testing.

### Data collection

Physicians screened potential cases for SARI or meningitis at hospital admission, after obtaining verbal consent, or proxy consent (for children), physicians reviewed patients’ medical records to obtain demographic information, past medical history including records on vaccinations, antibiotics use before hospitalization, clinical signs and symptoms, and radiographic results on a structured case report form. After discharge, physicians were also requested to update the case report form to complete data about treatment, disease outcomes and laboratory testing results. Patients were followed up to collect outcomes at 30 days after discharge.

### Specimen collection and testing

For SARI patients, nurses collected whole blood specimens and urine specimens (≥18 years) within two hours of enrollment or before the use of antibiotics following standardized procedures. Whole blood specimens (10ml for patients aged >15 years; 4 ml for those aged 7–14 years; 3 ml for those aged 3–7 years; 1 ml for those aged <3 years) were collected and then transferred to a hospital clinical laboratory within two hours to culture by BD-9050 automated culture system for *Sp*. For all SARI patients aged >18 years, nurses collected 10 ml of urine to detect *Sp* antigen. Urine specimens were tested by the Binax NOW *Streptococcus pneumoniae* urinary antigen test per the manufacturer’s instructions. For meningitis patients, the collection and testing of blood samples was the same as for SARI patients. Cerebrospinal fluid (CSF) was obtained within two hours of enrollment or before initiation of antibiotic treatment following standard procedures for lumbar puncture; 40 µl of CSF was inoculated into bacterial culture within one hour of collection. For *Sp* isolates, PCR was used to determine serotype using previously published methods[8].

### Data analysis

Data were entered into Epidata software (version 3.1) and imported into SPSS (v17.0, SPSS, Chicago, IL, USA) for analysis. To describe baseline characteristics, clinical, treatment, complication, and outcome, we conducted frequency analyses for categorical variables and estimated median and interquartile ranges (IQRs) for continuous variables. Patients with laboratory-confirmed *Sp* were divided into age groups (<5 years, 5–64 years group, and aged ≥65 years) and compared using chi-squared test and Fisher’s exact test to compare categorical variables, and *t*-test to compare continuous variables; all statistical tests were two-sided with *p*<0.05.

### Human subjects review

This project was approved by the ethical review committees at the Chinese Center for Disease Control and Prevention (China CDC, Beijing, China) and the U.S. Centers for Disease Control and Prevention (US CDC, Atlanta, GA, USA). In response to the 2009 H1N1 pandemic, in October 2009 China’s Ministry of Health issused the national surveillance protocal for SARI and authorized participating hospitals to collect individual case data and specimens from SARI patients. Therefore, for SARI patients in this study, participation only required patients or their parent/guardian to provide brief verbal consent. Blood culture and lumbar puncuture for suspected meningitis are the routine clinical practice in hospitals, consent in China for such procedures is almost always verbal, not written.

## Results

A total of 22,608 hospitalized patients in the four surveillance hospitals met either the SARI or meningitis case definitions from April 5, 2010 to September 30, 2012. Two hundred and thirty three (n = 233) refused to participate. We enrolled 22,375 total patients: 22,202 (99%) SARI and 173 (1%) meningitis.

### Patient characteristics

Twelve thousand eight hundred and forty two (12,842, 58%) SARI patients and 98 (57%) meningitis patients were male, and the median age was 2 years (interquartile range: 1–4) and 28 years (interquartile range: 8–46) for SARI and meningitis, respectively. Children aged < 5 years accounted for 79% of SARI patients while meningitis patients were most often aged 15–49 years (46%). About 1,316 (6%) SARI patients and 15 (9%) meningitis patients had at least one chronic disease, including hypertension (2%), chronic obstructive pulmonary disease (1%), chronic bronchitis (1%) and asthma (1%); hypertension (4%) and asthma (1%) were the chronic diseases most common in meningitis patients. Additionally, 19 SARI and 3 meningitis patients were pregnant.

Compared with meningitis patients, SARI cases were more frequently obese (21% vs 4%, p<0.001). Few patients were vaccinated with 7-valent pneumococcal conjugate vaccine (PCV 7) (3%), 23-valent pneumococcal polysaccharide vaccine (4%), and seasonal influenza vaccine (12%). (S1 Table)

### Laboratory testing

**Fig 1:**
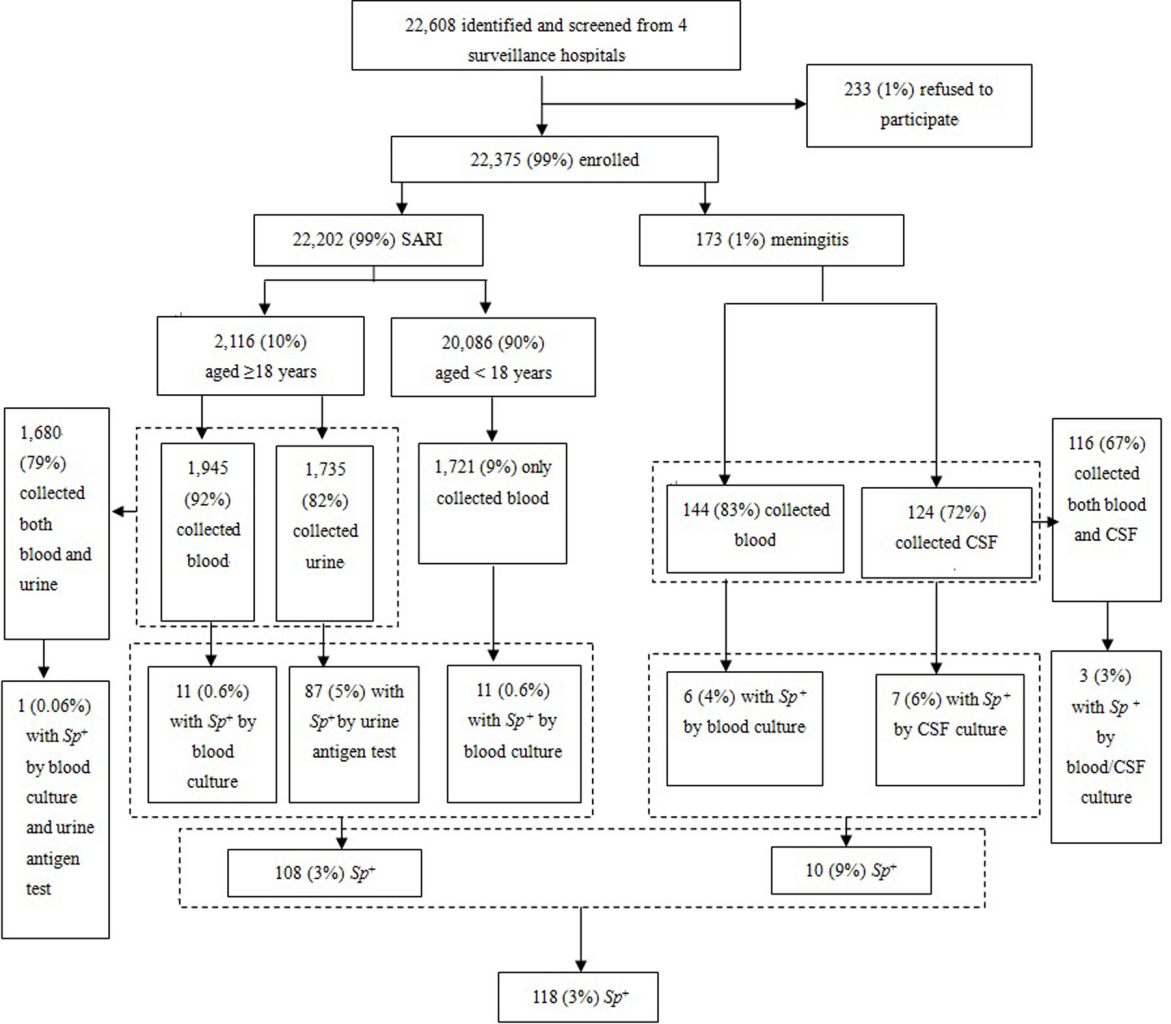
Enrollment of patients with SARI and meningitis, and results of laboratory testing for *Sp* infection in Jingzhou, China from April 2010 to September 2012. CSF, cerebrospinal fluid; SARI, severe acute respiratory infection; *Sp*, *Streptococcus pneumoniae*.

Overall, 118 (3%, 118/3868) patients with either SARI or meningitis were *Sp* positive: 32 (0.8%, 32/3819) *Sp* from blood/CSF culture and 87 (5%, 87/1735) *Sp* from urine antigen testing. (Fig 1). Among 2,116 SARI patients aged ≥18 years, blood culture was performed for 1,945 (92%), urine antigen testing for 1,735 (82%), and both methods for 1,680 (79%). *Sp* was recovered from 11 blood cultures (0.6%, 11/1945), and urine antigen testing detected *Sp* in 87 (5%, 87/1735); only 1 patient was *Sp* positive by both blood culture and urine antigen test. Among the 20,086 SARI patients aged <18 years, only 1,721 (9%) patients were tested by blood culture (other patients were not collected blood), and *Sp* was recovered from 11 (0.6%). Among the 173 meningitis patients, 144 (83%) patients were tested by blood culture, 124 (72%) patients by CSF culture, and 116 (67%) by both. *Sp* was recovered from 6 blood cultures (4%, 6/144), and 7 CSF cultures (6%, 7/124); three patients had both blood and CSF cultures positive for *Sp*.

### Characteristics of patients with *Sp* infection

Of the 118 patients with *Sp* infection, 57% were aged ≥65 years. Cough (85%), temperature ≥38°C (65%), and sputum production (60%) were the most common symptoms and signs. None of the patients with confirmed *Sp* infection were vaccinated with PCV 7, 23-valent pneumococcal polysaccharide vaccine, or seasonal influenza vaccine. Nearly 100% patients received antibiotics during hospitalization. Forty patients (34%) were treated with corticosteroid, and 8 (80%) were meningitis cases. 56 patients (48%) were also treated with oxygen treatment. Eighteen *Sp* patients were admitted to an intensive care unit. Two patients (pneumonia case) died during hospitalization, and no additional patients died within 30 days after discharge. (Table 1)

**Table 1:** Characteristics of patients with invasive *Sp*^+^ infection from four surveillance hospitals in Jingzhou, China, April 2010 to September 2012.

Characteristic	All *Sp* patients (N = 118) [n, (%)]	Aged<5 years (N = 9) [n, (%)]	5≤Aged<65 years (N = 42) [n, (%)]	Aged≥65 years (N = 67) [n, (%)]
Male sex	70 (59)	5 (56)	24 (57)	41 (61)
Age, Median [IQR, years]	68 [46–78]	2 [1–3]	48 [31–59]	77 [71–82]
Age group				
<5 year	9 (8)	9 (64)	-	-
5–17 years	5 (4)	-	5 (36)	-
18–49 years	16 (14)	-	16 (15)	-
50–64 years	21 (18)	-	21 (20)	-
≥ 65 years	67 (57)	-	-	67(64)
Underlying chronic medical conditions				
Any	59 (50)	0 (0)	13 (31)	46 (69)
Chronic bronchitis	20 (17)	0 (0)	3 (7)	17 (25)
Hypertension	20 (17)	0 (0)	3 (7)	17 (25)
Chronic obstructive pulmonary disease	13 (11)	0 (0)	2 (5)	11 (16)
Cardiovascular disease	13 (11)	0 (0)	0 (0)	13 (13)
Asthma	11 (9)	0 (0)	3 (7)	8 (12)
Diabetes	6 (5)	0 (0)	0 (0)	6 (6)
Renal dysfunction	1 (0.8)	0 (0)	0 (0)	1 (1)
Symptoms and signs at hospital admission				
Cough	100 (85)	6 (67)	31 (74)	63 (94)
Temperature ≥38°C	77 (65)	9 (100)	29 (69)	39 (58)
Sputum Production	71 (60)	1 (11)	19 (45)	51 (76)
Sore throat	13 (11)	0 (0)	10 (24)	3 (5)
Documented tachypnea	13 (11)	0 (0)	5 (12)	8 (12)
Runny nose	9 (8)	2 (22)	2 (5)	5 (8)
Difficulty breathing	9 (8)	1 (11)	4 (10)	4 (6)
Chest pain	7 (6)	0 (0)	4 (10)	3 (5)
Hemoptysis	4 (3)	0 (0)	2 (5)	2 (3)
Diarrhea	3 (3)	0 (0)	2 (5)	1 (2)
Disturbance of consciousness	3 (3)	1 (11)	2 (5)	0 (0)
Vomiting	2 (2)	0 (0)	1 (2)	1 (2)
Abdominal pain	1 (0.8)	0 (0)	1 (2)	0 (0)
Time interval				
From illness onset to hospital admission [IQR, days]	4 [1–7]	2 [1–5]	4 [2–7]	4 [1–7]
Length of stay in hospital [IQR, days]	10 [6–15]	6 [4–8]	11 [6–18]	11 [6–15]
Treatment				
Received antiviral drugs	2 (2)	0 (0)	2 (5)	0 (0)
Received antibiotics during hospitalization	117 (99)	9 (100.0)	41 (98)	67 (100)
Received antibiotics before hospitalization	45/89 (51)	2/4 (50)	20/32 (63)	23/53 (43)
Received corticosteroid during hospitalization	40 (34)	4 (44)	11 (26)	25 (37)
Received oxygen treatment	56 (48)	2 (22)	13 (31)	41 (61)
Received mechanical ventilation	5 (4)	1 (11)	0 (0)	4 (6)
Complications				
Respiratory failure	6 (5)	0 (0)	1 (2)	5 (8)
Cardiac failure	4 (3)	0 (0)	0 (0)	4 (6)
Hepatic dysfunction	2 (2)	0 (0)	2 (5)	0 (0)
Renal dysfunction	2 (2)	0 (0)	0 (0)	2 (3)
Neurologic disorders	2 (2)	0 (0)	2 (5)	0 (0)
ARDS	1 (0.8)	0 (0)	0 (0)	1 (2)
Clinical severity				
Pneumonia	84 (72)	4 (44)	27 (64)	53 (79)
Meningitis	10 (8)	2 (22)	8 (19)	0 (0)
ICU	18 (15)	2 (22)	7 (17)	9 (13)
Death	2 (2)	0 (0)	0 (0)	2 (3)

ARDS, acute respiratory distress syndrome; ICU, intensive care unit; IQR, interquartile range; SARI, severe acute respiratory infection; *Sp*, *Streptococcus pneumoniae*.

SARI patients with laboratory confirmation of *Sp* infection were older (median 70 years versus 23 years), and more often had at least one chronic medical condition (52% versus 29%), including chronic bronchitis, hypertension, chronic obstructive pulmonary disease, cardiovascular disease and asthma (p<0.05 for those comparisons) when compared with *Sp*-negative SARI patients. Patients with *Sp* detected were also more likely to have cough (p<0.001) and sputum production (p<0.001). These patients were less likely to have received seasonal influenza vaccine (p = 0.010) or to have received antibiotics before hospitalization (p = 0.048). During hospitalization, patients with *Sp* detected more often had pneumonia or respiratory failure diagnosed, more often received oxygen treatment and mechanical ventilation (Table 2), and had longer hospital stays (p = 0.003)

**Table 2:** Characteristics of SARI patients with *Sp*^+^ or *Sp*^-^ from four surveillance hospitals in Jingzhou, China, April 2010 to September 2012.

Characteristic	*Sp* Detected (N = 108) [n, (%)]	*Sp* Not Detected (N = 3613) [n, (%)]	*p-*Value^*^
Male sex	63 (58)	2167 (60)	0.734
Age, Median [IQR, years]	70 [56–78]	23 [3–64]	**<0.001**
Age group			**<0.001**
<5 year	7 (7)	1178 (33)	
5–17 years	4 (4)	532 (15)	
18–49 years	10 (9)	544 (15)	
50–64 years	20 (19)	518 (14)	
≥ 65 years	67 (62)	841 (23)	
Underlying chronic medical conditions			
Any	56 (52)	1039 (29)	**<0.001**
Chronic bronchitis	20 (19)	249 (7)	**<0.001**
Hypertension	19 (18)	388 (11)	**0.024**
Chronic obstructive pulmonary disease	13 (12)	255 (7)	**0.048**
Cardiovascular disease	13 (12)	214 (6)	**0.009**
Asthma	11 (10)	104 (3)	**0.001**
Diabetes	6 (6)	101 (3)	0.131
Renal dysfunction	1 (1)	43 (1)	0.795
Symptoms and signs at hospital admission			
Cough	98 (91)	2720 (75)	**<0.001**
Temperature ≥38°C	69 (64)	1946 (54)	0.112
Sputum Production	70 (65)	1312 (36)	**<0.001**
Documented tachypnea	13 (12)	260 (7)	0.057
Sore throat	12 (11)	469 (13)	0.569
Runny nose	9 (8)	343 (10)	0.685
Difficulty breathing	8 (7)	161 (5)	0.180
Chest pain	7 (7)	128 (4)	0.144
Hemoptysis	4 (4)	77 (2)	0.313
Time interval			
From illness onset to hospital admission [IQR, days]	4 [1–7]	4 [1–7]	0.385
Length of stay in hospital [IQR, days]	10 [6–15]	7 [5–11]	**0.003**
Vaccination			
PCV7	0 (0)	16/1636 (1)	0.090
PPV23	0 (0)	24/1943 (1)	0.073
Seasonal influenza vaccine	0 (0)	95/2061 (5)	**0.010**
Treatment			
Received antiviral drugs	2 (2)	19 (1)	0.148
Received antibiotics during hospitalization	108 (100)	3586 (99)	0.198
Received antibiotics before hospitalization	42/83 (51)	1609/2594 (62)	**0.048**
Received corticosteroid during hospitalization	32 (30)	786 (22)	0.051
Received oxygen treatment	50 (47)	758 (21)	**<0.001**
Received mechanical ventilation	4 (8)	31 (4)	**0.021**
Complications			
Respiratory failure	6 (6)	79 (2)	**0.049**
Cardiac failure	4 (4)	59 (2)	0.153
Renal dysfunction	2 (2)	26 (1)	0.255
ARDS	1 (1)	21 (1)	0.670
Hepatic dysfunction	0 (0)	42 (1)	0.115
Neurologic disorders	0 (0)	19 (1)	0.290
Clinical severity			
Pneumonia	83 (77)	1173 (33)	**<0.001**
ICU	12 (11)	0 (0)	0.628
Death	2 (2)	27/3612 (1)	0.272

*The P-values are comparisons between “*Sp* detected” and “*Sp* not detected”.

ARDS, acute respiratory distress syndrome; ICU, intensive care unit; IQR, interquartile range; PCV7, 7-valent pneumococcal conjugate vaccine; PPV23, 23-valent pneumococcal polysaccharide; SARI, severe acute respiratory infection; *Sp*, *Streptococcus pneumoniae*.

Laboratory confirmed *Sp* meningitis patients more frequently had liver dysfunction (p = 0.019), an elevated temperature ≥38°C (p = 0.047), received corticosteroids during their hospital stay (p = 0.039), and were admitted to the ICU during hospitalization (p = 0.013) (Table 3) when compared with *Sp* negative meningitis patients.

**Table 3:** Characteristics of meningitis patients with *Sp*^+^ or *Sp*^-^ from four surveillance hospitals in Jingzhou, China, April 2010 to September 2012.

Characteristic	*Sp* Detected (N = 10) [n, (%)]	*Sp* Not Detected (N = 142) [n, (%)]	*p-*Value^*^
Male sex	7 (70)	79 (56)	0.367
Age, Median [IQR, years]	33 [13–44]	32 [14–49]	0.648
Age group			0.263
<5 year	2 (20)	18 (13)	
5–17 years	1 (10)	26 (18)	
18–49 years	6 (60)	65 (46)	
50–64 years	1 (10)	24 (17)	
≥ 65 years	0 (0)	9 (9)	
Underlying chronic medical conditions			
Any	3 (30)	12 (8)	0.063
Hypertension	1 (10)	5 (4)	0.384
Liver dysfunction	1 (10)	0 (0)	**0.019**
Stroke	0 (0)	2 (1)	0.601
Asthma	0 (0)	2 (1)	0.601
Symptoms and signs at hospital admission			
Temperature ≥38°C	8 (80)	69 (49)	**0.047**
Nuchal rigidity	7 (70)	66 (47)	0.146
Kernig sign	5 (50)	30 (21)	0.053
Disturbance of consciousness	3 (30)	23 (16)	0.297
Brudzinski's sign	2 (20)	15 (11)	0.400
Twitch	2 (20)	10 (7)	0.204
Cough	2 (20)	30 (21)	0.932
Sputum Production	1 (10)	9 (6)	0.672
Sore throat	1 (10)	17 (12)	0.849
Difficulty breathing	1 (10)	2 (1)	0.161
Diarrhea	1 (10)	3 (2)	0.234
Vomiting	1 (10)	18 (13)	0.799
Abdominal pain	1 (10)	4 (3)	0.309
Time interval			
From illness onset to hospital admission [IQR, days]	4 [2–20]	4 [1–7]	0.279
Length of stay in hospital [IQR, days]	26 [8–29]	12 [6–19]	0.089
Vaccination			
PCV7	0 (0)	1/41 (2)	0.699
PPV23	0 (0)	1/43 (2)	0.750
Seasonal influenza vaccine	0 (0)	1/78 (1)	0.113
Treatment			
Received antiviral drugs	0 (0)	0 (0)	-
Received antibiotics during hospitalization	9 (90)	130 (92)	0.868
Received antibiotics before hospitalization	3/6 (50)	41/90 (46)	0.956
Received corticosteroid during hospitalization	8 (80)	67 (47)	**0.039**
Received oxygen treatment	6 (60)	55 (39)	0.190
Received mechanical ventilation	1 (10)	2 (1)	0.161
Complications			
Neurologic disorders	2 (20)	19 (13)	0.577
Hepatic dysfunction	2 (20)	5 (4)	0.062
Shock	0 (0)	1 (1)	0.712
Tympanitis	0 (0)	0 (0)	-
Clinical severity			
ICU	6 (60)	0 (0)	**0.013**
Death	0 (0)	1 (1)	0.712

*The P-values are comparisons between “*Sp* detected” and “*Sp* not detected”.

ICU, intensive care unit; IQR, interquartile range; PCV7, 7-valent pneumococcal conjugate vaccine; PPV23, 23-valent pneumococcal polysaccharide; SARI, severe acute respiratory infection; *Sp*, *Streptococcus pneumoniae*.

### Distribution of serotype

**Fig 2:**
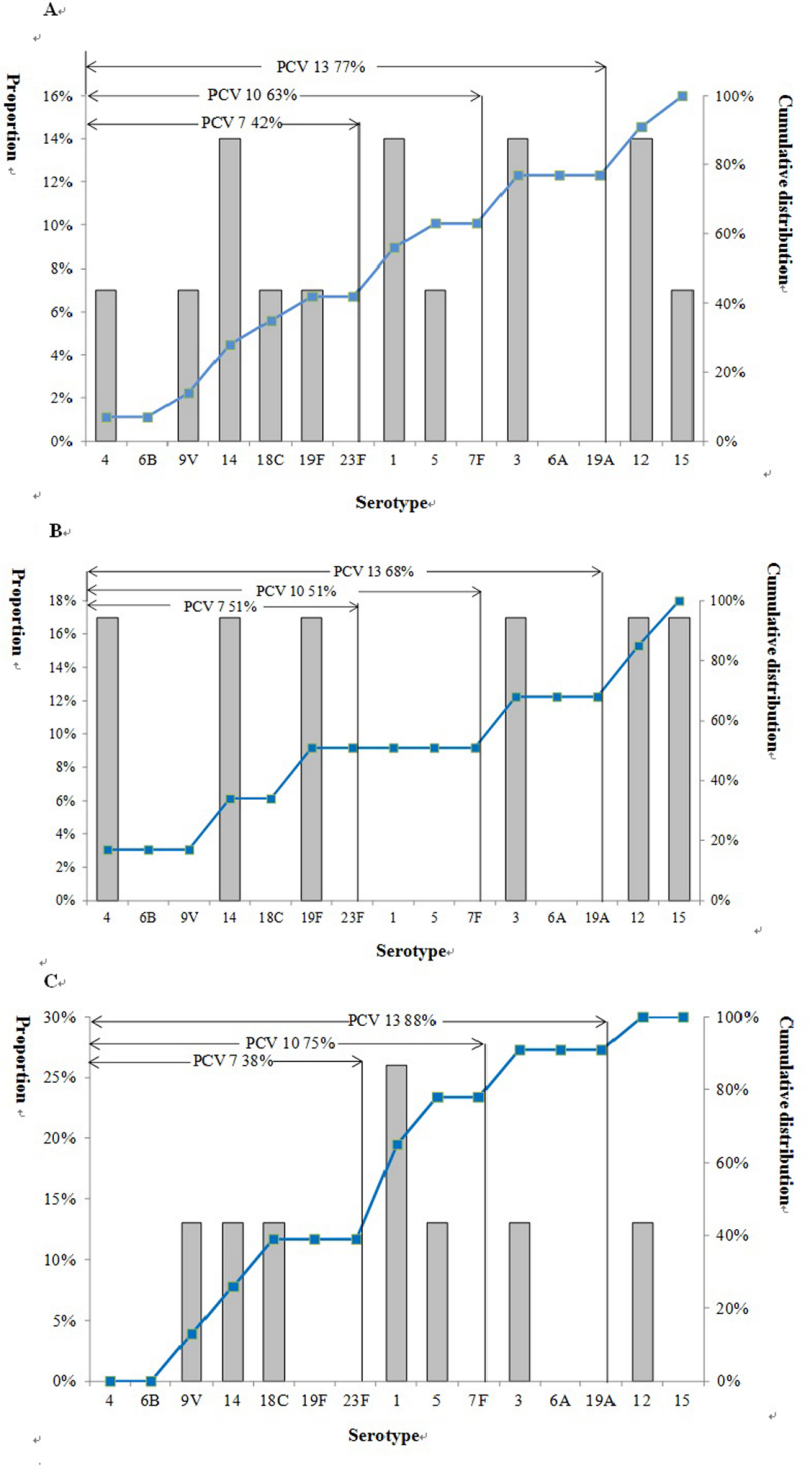
Proportion and cumulative distribution of serotype from *Sp*^+^ patients in Jingzhou, China from April 2010 to September 2012. Panel A: All *Sp*^+^ patients. Panel B: *Sp*^+^ patients aged<5 years. Panel C: *Sp*^+^ patients aged≥5 years.

Of 16 isolates serotyped, 2 were non-typeable, 2 each were 14, 12, 3 and 1, and one each was serotype 19F, 4, 5, 9V, 15 and 18C. For cases <5 years old, serotypes were 3, 4, 12, 14, 15 and 18; for cases 5–64 years old, serotypes 1, 12, 18C; and for cases ≥65, serotypes 3, 5, 9V and 14. The total coverage levels were 42%, 63% and 77% for PCV7, PCV10, and PCV13, respectively. Additionally, for persons aged <5 years, coverage levels were 51%, 51%, 68% for PCV7, PCV10 and PCV13. For persons aged ≥ 5 years, vaccine coverage was 38%, 75%, 88% for PCV7, PCV10 and PCV13. (Fig 2)

## Discussion

In this large, prospective study in China, *Sp* was infrequently identified as the cause of pneumonia and meningitis, and few patients were vaccinated against *S*p infection.

Multiple studies have been published in China documenting *Sp* as a common cause of respiratory infection in children and adults, but most relied on the use of non-sterile specimens, such as oro-pharyngeal or naso-pharyngeal swabs[5, 9–12]. Our study, in contrast, is the first large study in China to prospectively enroll patients with a broad case definition of severe acute respiratory infection or meningitis and to test specimens from sterile sites. *Sp* is considered the most common cause of bacterial community acquired pneumonia (CAP)[13], and may cause 30–50% of pneumonia requiring hospitalization in Europe and United States[14]. Although 72% of patients with *Sp* detected in our study were diagnosed with pneumonia, only 7% of patients diagnosed with pneumonia had *Sp* detected. *Sp* is also a common cause of meningitis globally[15], and was the most common cause of bacterial meningitis in one previous population-based study in China[16]. Nevertheless, *Sp* was only identified in 6% of patients who met our case definition of meningitis.

The low rate of *Sp* detection in our study may be due to the expansive case definition. First, we intentionally enrolled a broad cross-section of patients with severe illness, which resulted in many patients in whom *Sp* infection was unlikely to be detected, such as adults aged 18–64 years. Second, over 50% of patients received antibiotics before hospitalization, increasing the likelihood that bacterial cultures of blood and CSF would be negative[17–20]. Third, we embedded the study within routine clinical practice, which could have limited the quality of specimen collection and testing. The optimum time for specimen collection is actually before symptoms occur[21], but in our study, specimens were not collected until hospitalization, which occurred a median of 2 days after symptom onset. Lastly, the methods to detect pneumococcus, such as real-time PCR or Binax NOW® antigen test, can dramatically increase the detection of pneumococcus, especially in thoese already having received antibiotics. Although real–time PCR is a more sensitive method of detecting invasive pneumococcal disease, bacterial culture is the standard diagnostic method that has been used in local hospitals for years and there were insufficient funds to support this method.

WHO and ACIP recommend the use of conjugate pneumooccal vaccine to prevent invasive disease in children and the elderly[2, 22]. We found that no patients with *Sp* detected reported previous *Sp* vaccination. *Sp* vaccination is uncommon in China, because it is not included in the national immunization program for children or adults, to the relatively low awareness of the disease, and to the high out-of-pocket charges for *Sp* vaccine[23]. In our study, the median age of patients with *Sp* was 68 years, consistent with studies indicating the elderly are at high risk for invasive pneumococcal disease and can benefit from vaccination[24].

Despite their age and the frequency of comorbid medical conditions, few patients with *Sp* died in our study. There are other reasons such as prompt and supportive medical care, but one possibility is that early, widespread use of antibiotics prevented death[25]. We found that PCV13, which has recently been licensed in China, would have covered 77% of the *Sp* infections identified detected in our study, including 68% of those in people aged <5 years and 88% in those aged ≥5 years. WHO recommends that pneumococcal conjugate vaccine be included in national childhood immunization programs, but does not make a similar recommendation for the elderly[2]. Given the population-level and individual harms associated with widespread antibiotic use, the documented benefits of pneumococcal vaccination in other settings[1, 21, 26, 27], and the expected serotype coverage of PCV13 in China, further work is needed to expand access to pneumococcal vaccination in China, both among children and potentially among the elderly.

In China, inappropriate use of antibiotics is a widespread and serious problem[28, 29]. In our study, over 50% of enrolled patients received antibiotic treatment before hospitalization, and nearly 100% of enrolled patients received antibiotics during hospitalization, even though few had a documented bacterial infection. Widespread use of antibiotics may increase resistance and lead to individual health complications. China has recently begun programs to promote judicious prescribing of antibiotics and to restrict pharmacies from dispensing antibiotics without a doctor’s prescription. Additionally, corticosteroid treatment has been used widely as adjunctive treatment for adults with bacterial meningitis[30]. About 80% of meningitis patients with confirmed *Sp* infection received corticosteroid treatment. Corticosteroid treatment has been independently associated with favorable outcomes and survival in patients with pneumococcal and non-pneumococcal meningitis[31, 32].

In addition to the limitations noted above, our study was also limited to only one city, did not enroll outpatients, and relied on routinely collected data, albeit prospectively, from medical records. It is possible that we, therefore, under-estimated incidence and disease burden. Nevertheless, our study adds to the limited literature about *Sp* infections in China and provides further data to support the potential benefits of *Sp* vaccination among children and the elderly in China. Furthermore, the characterization of *Sp* isolates and infections in this study can contribute to more rapid detection and control of *Sp* outbreaks in China, and thereby contribute to enhanced global health security.

## Supporting information

S1 Table Characteristics of hospitalized SARI and meningitis patients in Jingzhou, China, from April 2010—September, 2012. (DOCX)
