# Supplementary material for: Invasive Streptococcus pneumoniae infection among hospitalized patients in Jingzhou city, China, 2010-2012
Source: PLoS One. 2018 Aug 20;13(8):e0201312. doi: 10.1371/journal.pone.0201312 (PMC6101356; doi:10.1371/journal.pone.0201312)
Supplement: S1 Table — (DOCX) [file pone.0201312.s001.docx]

**S1 Table. Characteristics of hospitalized SARI and meningitis patients in Jingzhou, China, from April 2010 - September, 2012.**

| **Characteristic** | **SARI patients**  **(N=22,202)**  **[n, (%)]** | **Meningitis patients (N=173)**  **[n, (%)]** |
| --- | --- | --- |
| Male sex | 12,842 (58) | 98 (57) |
| Age, Median [IQR, years] | 2 (1-4) | 28 (8-46) |
| Age group |  |  |
| <5 year | 17,442 (79) | 31 (18) |
| 5-14 years | 2,551 (12) | 29 (17) |
| 15-49 years | 686 (3) | 79 (46) |
| 50-64 years | 563 (3) | 25 (15) |
| ≥65 years | 960 (4) | 9 (5) |
| Underlying chronic medical conditions |  |  |
| Any | 1,316 (6) | 15 (9) |
| Hypertension | 428 (2) | 6 (4) |
| Chronic bronchitis | 296 (1) | 1 (0.6) |
| Cardiovascular disease | 291 (1) | 1 (0.6) |
| Chronic obstructive pulmonary disease | 279 (1) | 1 (0.6) |
| Asthma | 205 (1) | 2 (1) |
| Diabetes | 110 (0.5) | 1 (0.6) |
| Renal dysfunction | 50 (0.2) | 1 (0.6) |
| Neurological disease | 1(0.0) | 0 (0) |
| Pregnancy | 19/617 (3) | 3/13 (23) |
| Obesity^§^ | 1,381/6,656 (21) | 5/139 (4) |
| Vaccination |  |  |
| PCV7 | 390/12,640 (3) | 1/57 (2) |
| PPV23 | 571/12,748 (4) | 2/59 (3) |
| Seasonal influenza vaccine | 1,758/14,176 (8) | 4/97 (4 ) |

§Obesity was measured by following Chinese BMI criteria: BMI ≥28 in adults, or BMI cut–off values for children aged 2-17 years. BMI was not calculated for young children aged< 2 years or pregnant women.

BMI, Body-Mass Index; IQR, interquartile range; PCV7, 7-valent pneumococcal conjugate vaccine; PPV23, 23-valent pneumococcal polysaccharide vaccine; SARI, severe acute respiratory infection.
